# Genome wide identification and functional characterization of strawberry pectin methylesterases related to fruit softening

**DOI:** 10.1186/s12870-019-2225-9

**Published:** 2020-01-08

**Authors:** Cheng Xue, Si-Cong Guan, Jian-Qing Chen, Chen-Jin Wen, Jian-Fa Cai, Xu Chen

**Affiliations:** 1 College of Horticulture, Fujian Agriculture and Forestry University, Fuzhou, 350002 Fujian China; 2 Haixia Institute of Science and Technology, Horticultural Plant Biology and Metabolomics Center, Fujian Agriculture and Forestry University, Fuzhou, 350002 China

**Keywords:** Pectin methylesterases (PMEs), Strawberry, Evolution, Fruit softening

## Abstract

**Background:**

Pectin methylesterase (PME) is a hydrolytic enzyme that catalyzes the demethylesterification of homogalacturonans and controls pectin reconstruction, being essential in regulation of cell wall modification. During fruit ripening stage, PME-mediated cell wall remodeling is an important process to determine fruit firmness and softening. Strawberry fruit is a soft fruit with a short postharvest life, due to a rapid loss of firm texture. Hence, preharvest improvement of strawberry fruit rigidity is a prerequisite for extension of fruit refreshing time. Although PME has been well characterized in model plants, knowledge regarding the functionality and evolutionary property of *PME* gene family in strawberry remain limited.

**Results:**

A total of 54 *PME* genes (*FvPMEs*) were identified in woodland strawberry (*Fragaria vesca* ‘Hawaii 4’). Phylogeny and gene structure analysis divided these *FvPME* genes into four groups (Group 1–4). Duplicate events analysis suggested that tandem and dispersed duplications effectively contributed to the expansion of the PME family in strawberry. Through transcriptome analysis, we identified *FvPME38* and *FvPME39* as the most abundant-expressed *PME*s at fruit ripening stages, and they were positively regulated by abscisic acid. Genetic manipulation of *FvPME38* and *FvPME39* by overexpression and RNAi-silencing significantly influences the fruit firmness, pectin content and cell wall structure, indicating a requirement of PME for strawberry fruit softening.

**Conclusion:**

Our study globally analyzed strawberry pectin methylesterases by the approaches of phylogenetics, evolutionary prediction and genetic analysis. We verified the essential role of *FvPME38* and *FvPME39* in regulation of strawberry fruit softening process, which provided a guide for improving strawberry fruit firmness by modifying PME level.

## Background

Cell wall is a structural layer surrounding the plant cell membrane, providing protection and strength for plant growth. The construction, differentiation, maturation and degradation of cell wall creates a rigid but flexible outer surrounding for cell division, cell differentiation and multi-cellular organ patterning [1, 2]. The cell wall is composed of a cellulose-hemicellulose network with the cross-linked pectin [3]. Pectin is defined as a hetero-polysaccharides, predominantly containing homogalacturonan (HG), rhamnogalacturonan-I, rhamnogalacturonan-II, and xylogalacturonan components, contributing to wall porosity, wall hydration, and intercellular adhesion [4, 5].

Homeostasis of pectin is regulated by different kinds of pectin-modifying enzymes [6]. Pectin methylesterases (PME, EC 3.1.1.11) is one of the pectin-modifying enzymes, [7] which plays diverse roles in plant development [8]. PME catalyzes reactions on the demethylesterification of pectin to generate carboxyl groups during the release of methanol and hydrogen ions [9]. De-esterification of the major component, HG by PME leads to the generation of free carboxylic acid groups [9, 10]. A low level of HG methylesterification due to the low PME activity often results in an increase of wall rigidity, which influences diverse aspects of plant development, such as hypocotyl growth [11, 12], pollen tube elongation [13, 14], embryo development [15], and seed germination [16, 17]. In contrast, a high level of HG demethylesterification by a higher PME activity produces an opposite effect, that is, loosening of the cell wall. For example, application of high level PME onto the flower primordia induces the formation of ectopic primordia, which is caused by the loosening cell wall structure [18, 19].

Beside the above plant developmental processes, PME-controlled pectin modification is also involved in the regulation of fruit quality. During fruit ripening, a range of pectin-degrading enzymes are secreted into the cell wall, leading to the degradation of pectin polymers and decrease of pectin level [20]. The resultant fruit developmental process is called fruit softening. In strawberry (*F*. × *ananassa*, Duch. cv Aroma), PME activity has a close relationship with softening. It induces an abrupt increase of acid pectin during strawberry fruit ripening [21] and is reduced immediately after UV-C irradiation with the consequence of a firmer fruit [22]. *FaPE1* is specifically expressed in strawberry fruit, showing an increasing expression during ripening process up to a maximum at turning stage [23]. In tomato fruits, the silence of PME enzyme is associated with an increased level of soluble solids and decreased level of soluble polyuronides in cell walls, which results in the increase of fruit rigidity [24]. In apple fruit ripening, the plant hormone ethylene and low temperature significantly increase PME activity to accelerate fruit softening [25, 26]. Thus, PME-mediated cell wall modification is an essential process to control fruit quality and rigidity.

Genome wide identification of *PME* genes has been widely studied in many plant species, such as *Arabidopsis* [27], rice (*O. sativa* subsp. *Japonica* cv.) [28], poplar (*Populus spp*.) [29], flax [30], and Asiatic cotton (*Gossypium arboretum*) [31]. All PME genes from those species contain a catalytically active zone PME domain, and some of them also comprise a pectin methylesterase inhibitor (PMEI) domain [27, 28, 30, 31]. With expression assay, those studies found some candidate *PME* genes which have tissue-specific expression patterns. For example, eight *PME*s from cotton showed fiber predominant expression in secondary wall thickening, which provided an important basis for further research on the functions of *PME*s in cotton fiber development [31]. Although PME has been well characterized in model plants, knowledge regarding the functionality and evolutionary property of *PME* gene family in Rosaceae plant species remain limited.

*Fragaria vesca*, the woodland strawberry, is emerging as a model plant of the Rosaceae plant species due to its small and sequenced genome, diploidy (2n = 14, 240 Mb genome) [32]. Strawberry fruit is a soft fruit with a short postharvest life, due to a rapid loss of firm texture. Hence, preharvest improvement of strawberry fruit rigidity is a prerequisite for extension of fruit refreshing time. In our study, we aim to improve strawberry fruit rigidity by manipulation of key cell wall degrading enzymes, PMEs, during fruit development. Firstly, through the global analysis of the genome sequence of strawberry *PME* (*FvPME*) genes, 54 unigenes were identified as candidate members of *FvPMEs*. Phylogenetics, gene structure, and predicted function were performed to characterize FvPMEs. Transcriptome analysis showed that *FvPME38* and *FvPME39* were particularly abundant in fruit ripening stage. Associated with the gradually decreased fruit firmness during ripening, transcripts of *FvPME38* and *FvPME39* were gradually increased. Further transient genetic manipulation of *FvPME38* and *FvPME39* in strawberry fruit by overexpression and silence approach supported the conclusion that *FvPME*s were required for the regulation of pectin content and fruit firmness. Our study provided a preliminary knowledge for improving strawberry fruit firmness by modification of PME enzymes.

## Methods

### Plant materials

The 7th generation inbred lines of *F. vesca* accession, namely Ruegen (Ru F7–4, red-fruited) were used as wild-types in this study [33]. The plants were grown in a greenhouse (16 h/8 h light conditions at 22 °C, at a relative humidity of 65%). The samples, used for RNA isolation, were frozen in liquid nitrogen immediately after collection and then stored at − 80 °C.

### Genome-wide identification of PME genes

The gene files of *Arabidopsis thaliana* were downloaded from TAIR (The *Arabidopsis* Information Resource, http://www.arabidopsis.org/). The gene files of *Fragaria vesca* (strawberry), *Malus domestica* (apple), *Prunus mume* (Chinese plum), *Prunus persica* (peach) and *Rosa chinensis* (rose) were downloaded from GDR database (Genome Database for Rosaceae: http://www.rosaceae.org/). The gene files of *Pyrus bretschneideri* (pear) were downloaded from the pear genome database (http://peargenome.njau.edu.cn/).

The Hidden Markov Model (HMM) profiles of PF01095 (PME domain) and PF04043 (PMEI domain) were downloaded from PFam database (http://pfam.sanger.ac.uk/), and the HMMER software package [34] was used to detect *PME* genes with the best domain e-value cutoff of 1e^− 10^. These sequences were regarded as potential *PME* genes. To validate the HMM search, these sequences of candidate *PME* genes were used as queries to search the NCBI non-redundant protein database through blastp program of GenBank, and only the results with the best hits (an e-value less than 1e^− 5^) of ‘pectin methylesterases’ and ‘pectin methylesterases inhibitor’ were used for the following study.

### Phylogenetics, gene structure and motif analyses

A rooted phylogenetic tree was constructed using MEGA X [35] with neighbor-joining (NJ) criteria and verified using the maximum likelihood (ML) method, and 1000 bootstrap replicates were performed based on the multiple alignments of the full-length amino acid sequences of all PME genes in *Arabidopsis thaliana*, *Fragaria vesca*, *Malus domestica*, *Pyrus bretschneideri*, *Prunus mume*, *Prunus persica* and *Rosa chinensis* using ClustalW [36]. Based on the alignments of CDS sequences with the corresponding full-length genomic sequences, the gene structures of the *FvPME* genes were displayed using an online website: Gene Structure Display Server (GSDS) [37]. Moreover, conserved motifs were detected in *FvPME* family members using the motif analysis tool Multiple Em for Motif Elicitation (MEME) [38] with the default parameters except for two: motif site distribution, any number of repetitions; maximum number of motifs, 30.

### Synteny analysis

We used a modified method to perform the synteny analysis, based on the method which was used previously in the Genome Duplication Database (PGDD) [39]. Firstly, a BLASTP alignment was carried out across the whole genome to identify the candidate homologous gene pairs (E < 1e^− 5^, top 5 matches). The gene candidates were then uploaded to the software MCScanX with the default parameters [40, 41] to identify syntenic chains. We also used MCScanX for further distinguishing the WGD/segmental, dispersed, proximal, and tandem duplication events of the PME gene family.

### Identification of cis-element on promoters of *FvPME* genes

Cis-elements on the 1.5 kb promoter sequences of *FvPME* genes were predicted by PlantCARE [42]. And the position of the cis-elements were displayed using an online website: Gene Structure Display Server (GSDS) [37].

### Gene expression analyses

The RNA-Seq data of different strawberry varieties was obtained from NCBI (Neinongxiang, PRJNA438551; Toyonoka, PRJNA394190; Camarosa, PRJEB12420; Sweet Charlie, PRJNA263114; Benihoppe, PRJNA473417; Yellow wonder, SRA065786). The heatmaps were plotted in R using the heatmap.2 function based on the logarithmically (log2) transformed reads per kilobase per million (RPKM) values of each PME gene.

### Texture analyses

TA.XT.plus Texture Analyser (Stable Micro Systems Ltd., Surrey, UK) along with the measuring probe P/5S (5 mm Spherical stainless steel, supplied with the Texture Analyser) were employed for texture determination. The system was equipped with texture profile analysis (TPA). Hardness was measured as the maximum penetration force (N) reached during tissue breakage. The maximum penetration force was set as 25 N. Other measurable parameters were: pretest speed 1 mm·s^− 1^; test speed 1 mm·s^− 1^ penetrating distance of 5 mm into the fruit. The measurement was triggered automatically at 0.04 N. The maximum force required for sample compression was calculated as an average of 10 measurements.

### Plasmid construction

The primers used for plasmid construction are listed in Additional file 2: Table S1. The coding region of *FvPME* genes were amplified from the cDNA of ‘Ruegen’ using PrimerSTAR® GXL DNA Polymerase (TaKaRa, Japan), sub-cloned into pDONR221, and then inserted into the binary vector pK7WGF2 using Gateway® Technology. For RNAi, the partial coding sequences of *FvPME38* (1–385 bp) and *FvPME39* (1-361 bp) were sub-cloned into pDONR221, and then inserted into the binary vector pB7GWIWG2. The correct fusion constructs were transferred into *Agrobacterium tumefaciens* strain GV3101 by the freeze–thaw method.

### Subcellular localization of FvPME

The leaves of 3-week-old *Nicotiana benthamiana* plants were infiltrated through their abaxial surfaces by *Agrobacterium* suspension (OD_600_ = 0.6). At 72 h post-infiltration, leaves were stained by FM4–64, and fluorescence signal was visualized using Zeiss LSM880 confocal microscope (Zeiss, Germany).

### Histological analysis

Different developmental stages of ‘Ruegen’ fruit were fixed in formalin–acetic acid–alcohol (FAA) fixative [formaldehyde solution: glacial acetic acid: 70% ethyl alcohol (v/v); 5: 5: 90, v/v] at 4 °C for 1 week. Samples were dehydrated in an ethanol series solution and embedded in paraffin. Cross-section slicing (10 μm) was performed by Leica RM2255 ultramicrotome (Leica Mikrosysteme, Germany) and stained by 1% (w/v) Toluidine Blue O. After staining, the sections were observed using Nikon SMZ18 microscope.

### Transiently transformed strawberry fruit flesh

Transiently transformed strawberry fruit flesh was carried out using agro-infiltration as previous described [43]. GV3101 strains which harbors *FvPME38* and *FvPME39* overexpression or RNAi constructs was infiltrated into the ‘Ruegen’ fruit flesh at 18 d after pollination (DAP) using syringes. Six plants or fruits were injected with each construct in triplicates. The transformed samples were placed in the dark at 22 °C overnight and then transferred to a phytotron (22 °C, 16-h of light and 8-h darkness) for 7 days before phenotype analysis.

### Cell wall pectin content

Measurement of cell wall pectin content was carried out according to the previous study [44]. The alcohol-insoluble cell wall materials of fruits were first isolated with 96% ethanol, weighed and hydrolysed by incubating samples in concentrated H_2_SO_4_. The uronic acid content was determined colorimetrically using a microplate spectrophotometer Multiskan GO 1510 (Thermo Fisher, USA). Galacturonic acid was used as a calibration standard, thus the fruit pectin content was expressed as galacturonic acid equivalents (GaE).

### Gene expression analysis by qRT-PCR

cDNA, used for quantitative reverse transcription–PCR (qRT-PCR) analysis, was synthesized using one-step genomic DNA removal and a cDNA synthesis kit (Transgen, China). qRT–PCR was performed using the MonAmp™ ChemoHS qPCR Mix (Monad, China). Primers are synthesized by Sangon Biotech Company (China) and shown in Additional file 2: Table S1. The analysis was performed using three biological samples and three technical repeats. Relative expression levels of each gene were normalized to internal control *Fvactin* and *FvGAPDH* by 2^−ΔΔCp^ algorithm [45].

### Hormonal treatment assay of strawberry fruits

The concentration of hormone solution is in accordance with previous study [46]. The concentration of stock solution was 5 mM in ethanol for nordihydroguaiaretic acid (NDGA) (Aladdin, China). The concentration of working solution for treatment were 100 μM for NDGA diluted in ddH_2_O. About 20 μL NDGA working solution was injected into the 15 DAP fruits.

### Statistical analysis

Statistical analysis was done by one-way ANOVA in the Graphpad prism 8.0 software (Graphpad Software, USA). Significant differences between groups were calculated using *P* < 0.05 in one-way ANOVA analysis, Tukey’s HSD post hoc test.

### Accession numbers

Sequence data in this article can be found at GenBank with the following accession numbers: FvPME38 (MK775554); FvPME39 (MK775555).

## Results

### Identification of PME genes in rosaceous plants

To identify PME gene sequence of Rosaceous plants, PME candidate genes were searched from Rosaceous plant species of *Fragaria vesca, Malus domestica, Pyrus bretschneideri, Prunus mume, Prunus persica* and *Rosa chinensis* genome according to two strategies: Hidden Markov Model search (HMMsearch) using the HMM profiles PF01095 (PME domain) and PF04043 (PMEI domain); BLASTP search using PME proteins from *Arabidopsis* as queries. As a result, 54, 78, 79, 57,66 and 53 PME genes were individually identified from *Fragaria vesca* (strawberry)*, Malus domestica* (apple)*, Pyrus bretschneideri* (pear)*, Prunus mume* (Chinese plum)*, Prunus persica* (peach) and *Rosa chinensis* (rose) (Additional file 2: Table S2), named as *FvPME*, *MdPME*, *PbPME*, *PmPME*, *PpPME*, and *RcPME* (Table 1). Based on the domain structures, these *PME* genes were classified into two sub-families: type I PME contains only PME domain, and type II PME contains both PME and PMEI domains (Additional file 2: Table S3).

**Table 1:** Summary of PME gene family in *Arabidopsis thaliana, Fragaria vesca, Malus domestica, Pyrus bretschneideri, Prunus mume, Prunus persica* and *Rosa chinensis*

Group	Arabidopsis	Strawberry	Apple	Pear	Chinese plum	Peach	Rose
Group1	35	29	40	38	24	23	27
Group2	10	5	11	9	8	10	6
Group3	2	2	4	8	4	3	2
Group4	19	18	23	24	21	30	18
Total	66	54	78	79	57	66	53

### Phylogenetics analysis

**Fig. 1:**
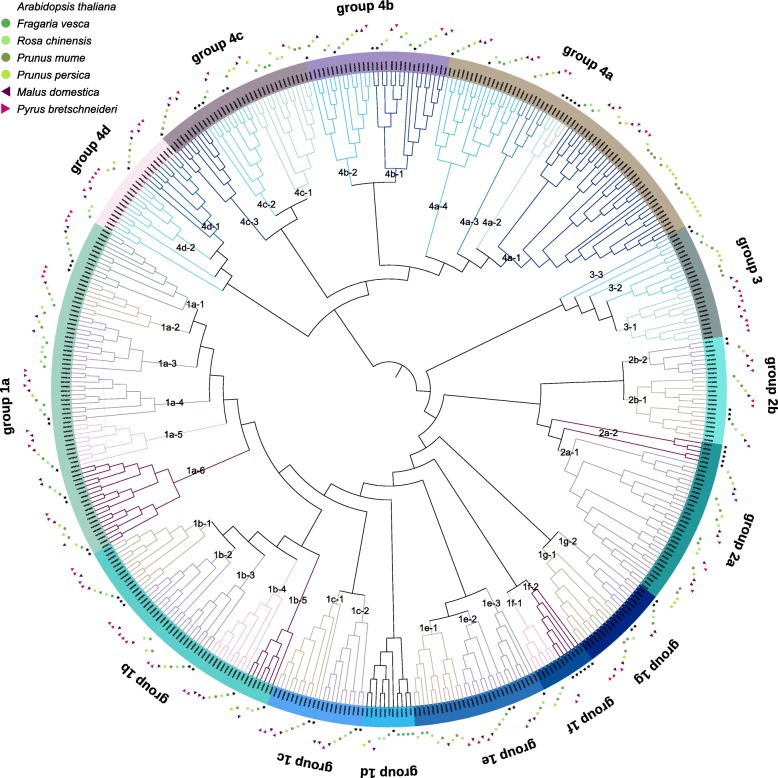
Maximum likelihood phylogenetic analysis of *PME* family in *Arabidopsis thaliana, Fragaria vesca, Malus domestica, Pyrus bretschneideri, Prunus mume, Prunus persica* and *Rosa chinensis*. The full-length sequences of PME proteins were aligned using ClustalX, and the phylogenetic tree was generated using MEGA X with the NJ method. Those PME genes were divided into four main groups, among them, groups 1, 2 and 4 could be further divided into serval sub-groups (groups 1a-1 g, 2a-2b and 4a-4d). Different sub-groups were labelled and distinguished with each other by different branch color. Furthermore, PME genes from different species were labelled with different shape types

To explore the evolutionary relationships among *Arabidopsis* and six Rosaceous plants, we constructed a phylogenetic tree using MEGA X based on the multiple sequence alignments of 66 *Arabidopsis* PMEs (*AtPME*) and 387 *PMEs* of Rosaceous plants. The phylogenetic tree showed that the PME genes was split into four groups, which were strongly supported by bootstrap values (Fig. 1). Based on the classification of *AtPMEs*, the 387 PME genes with PME and PMEI domains were clustered in groups 1–3, and the rest which only contain PME domain belong to group 4. In addition, groups 1, 2 and 4 were divided into serval sub-groups (groups 1a-1 g, 2a-2b and 4a-4d). Based on this classification, we found that most of the clades/subclades are consisted of genes both from the Rosaceous plants and *Arabidopsis*, suggesting that *AtPMEs* and *PME* genes of Rosaceous plants are evolved from a common ancestor. Meanwhile, we also identified species-specific PME subclades, such as 1a-2, 1a-3, 1b-2, 1e-1, 1f-1, 2a-1, 3–1, 3–2, 4a-1, 4a-3, 4c-1, and 4d-2 subclades (Fig. 1), implying an independent evolutionary event happened among Rosaceous plants. Interestingly, in group 3, *PME* genes from apple and pear were clustered in subclade 3–1, which were entirely isolated from the *PMEs* of strawberry, rose, peach, and Chineses plum species. Moreover, in species-specific subclade 4a-1, the gene number of *PMEs* from peach and Chineses plum were intensely expanded, suggesting a particular function of PMEs in peach and Chinese plum.

### Different duplication events control the expansion of PME genes in rosaceous plants

**Fig. 2:**
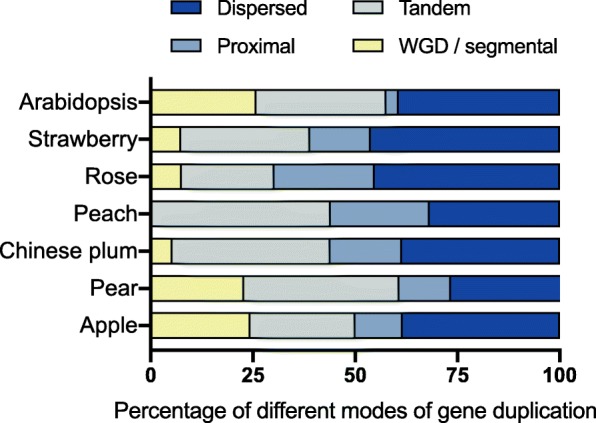
Comparison of different modes of gene duplication in seven species The y-axis shows the six species. The x-axis represents the percentage of different modes of gene duplication.

To further understand how PME genes are evolved, gene duplication events were investigated in *Arabidopsis* and six Rosaceous plant species. As shown in Fig. 2, dispersed gene duplication represents the major event of gene expansion, as it accounted for 39.4% (26 of 66), 46.3% (25 of 54), 38.5% (30 of 78), 38.6% (22 of 57) and 45.3% (24 of 53) of PME genes in *Arabidopsis*, woodland strawberry, apple, Chinese plum and rose, respectively. In contrast, tandem gene duplication occurred more frequently in pear and peach, which is accounted for 38% (30 of 79) and 43.9% (29 of 66) (Additional file 2: Table S4). Besides, WGD/segmental duplication is another main driving force for PME gene family expansion in pear, apple and *Arabidposis* (Fig. 2 and Additional file 2: Table S4). These results implied that different duplication events controlled PME family expansion in *Arabidopsis* and six Rosaceous species.

### Gene structure, conserved motif and physical distribution of *FvPME* genes

**Fig. 3:**
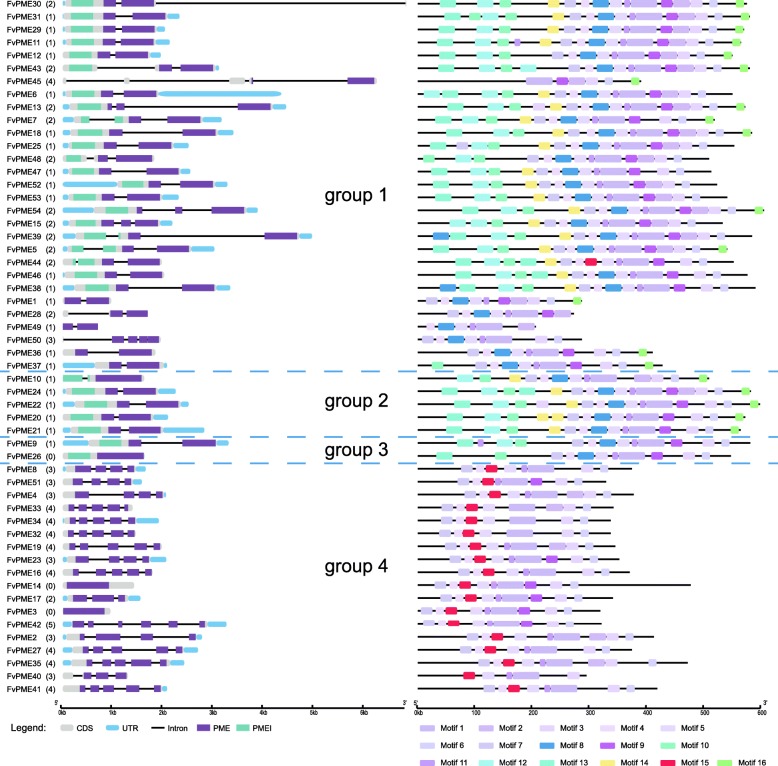
Analyses of gene structure and conserved motifs within each *PME* group in strawberry. **a**, Exon/intron structures of strawberry *PME* genes. White boxes represent the exons. Blue boxes represent UTRs. Black lines connecting two exons indicate introns. Sequence of purple boxes and green boxes code PME domain and PMEI domain, respectively. **b**, The distribution of conserved motif within each *PME* genes. Boxes in different colors represent different conserved motifs, and their relative position are displayed

As mentioned above, strawberry fruit is highly perishable. To improve strawberry fruit rigidity and extend fruit refreshing time, we first analyzed strawberry *PME* genes by bioinformatics and genetic analysis. Gene structure and conserved motif analysis provided further evidence to support the phylogenetic topology groupings of gene families. Gene structure analysis of *FvPMEs* showed that most members in groups 1–3 contain one or two introns which is located in the conserved position, while group 4 members contain three to four introns whose positions are varied (Fig. 3a). To search for the potential conserved motifs of *FvPMEs*, we applied MEME tool to analyze the sequences of 54 *FvPMEs*. A total of 16 conserved motifs were detectable, named as motif 1–16 (Fig. 3b and S1). Most of the motifs are conserved. Particularly, motifs 14 and 16 are only present in groups 1 and 2 members, motif 8 only appears in groups 1–3 members, and motif 15 was exclusively found in group 4 members. Those special motifs might contribute to the diverse function of *PME* genes from different groups.

**Fig. 4:**
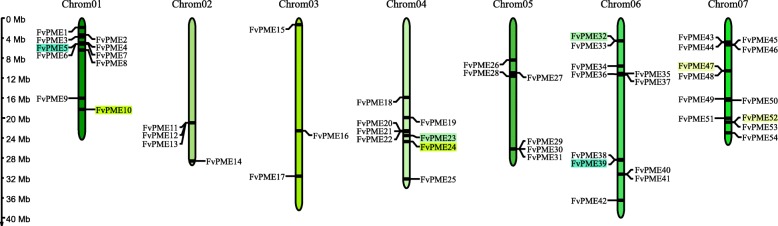
Chromosomal locations of *FvPME* genes. The chromosomal position of each *FvPME* genes was mapped according to the strawberry genome. The chromosome number is indicated at the top of each chromosome. Segmental duplicates represented by colored boxes, and tandem duplicates are marked by black sides

Regarding the physical genome distributions, 54 *FvPME*s are individually located in seven chromosomes. Among them, chromosome 7 contains the largest numbers of 12 *FvPME*s, chromosome 6 contains 11 members and chromosome 1 contains 10 members. In contrast, only three *FvPME*s are present on the chromosome 3 (Fig. 4). Interestingly, a higher density of *FvPME*s was found on the particular regions of chromosomes, such as the top of chromosome 1, and the bottom of chromosomes 7.

### Putative cis-element in *FvPME* gene promoters

To identify the possible gene responsiveness of *FvPMEs*, a 1.5 kb promoter region is captured and analyzed by PlantCARE (Additional file 2: Table S5). Bioinformatics analysis indicates that most of the *cis*-elements of *FvPMEs’* promoters belong to the responsive elements of plant hormones, abiotic (e.g., dehydration and salt) and biotic stress (Additional file 2: Table S5). Particularly, a large number of transcription factor binding sites were present on the promoter of *FvPME* genes (Additional file 1: Fig. S2). These findings implied that the transcriptional level of *FvPME* genes are variously controlled by various factors including phytohormones and environmental stimuli during plant developmental processes.

### Functional prediction of *FvPME* genes

In principle, homologous genes share similar gene structures and are clustered in the same clades, in which the genes possess similar functions [47]. Phylogenetic analysis allowed us to predict putative gene function of orthologous and paralogous *PME* genes in strawberry (Table 2). For example, group 1a is the biggest subgroup with diverse functions, involved in adventitious rooting, nematode defense and seedling development in *Arabidopsis* [48, 49], implying a similar role of *FvPME* in group 1a *FvPME38* and two *AtPME* genes (*AtPME9* and *AtPME28*) belong to subgroup 1 g-2. *AtPME 9* and *AtPME28* have been reported to participate in cell wall remodeling processes and response to abiotic stress [55, 56]. Possibly, the homologue strawberry gene *FvPME38* is also involved in cell wall regulation.

**Table 2:** Putative functions of strawberry PME

Subgroup	*Fragaria vesca*	*Arabidopsis thaliana*	Functions	Reference
1a-1	FvPME11	AtPME43	sensitive to chilling stress and brassinosteroid regulation	[48]
1a-6	FvPME39	AtPME29	involving in adventitious rooting / root hypersensitivity to zinc / nematode infection; seeding development	[49]
1b-3	FvPME47	AtPME18	Involved root development and in response to various stresses	[50]
1c-1	FvPME54	AtPME36	Promoting stomatal movement under heat stress	[51]
1c-2	FvPME18	AtPME17; AtPME37	response to biotic stress; regulating mechanical strength of the supporting tissue	[52, 53]
1f	FvPME15	AtPME32	root development	[54]
1 g-2	FvPME38	AtPME9; AtPME28	cell wall remodeling processes; response to abiotic stress	[55, 56]
2a	FvPME20 FvPME21 FvPME22	AtPME60	seed coat mucilage extrusion	[57]
2b-1	FvPME10 FvPME24	AtPME19 AtPME20	pollen tube growth	[58]
4a	FvPME51 FvPME8	AtPME10 AtPME50	Promote pollen tube growth; involved in plant immune responses	[59]
4b-1	FvPME16	AtPME64	Assisting in the liberation of pollen grains from tetrads during floral development.	[60–64]
4b-2	FvPME27	AtPME59	organ initiation and cell elongation	[65]
4c-2	FvPME19	AtPME31	response to salt stress	[66]

In addition, gene clusters with similar function were observed for other PME genes in subgroups 1c-1,1c-2 and 4c-2, among which the homologue genes from *Arabidopsis* (*AtPME36*, *AtPME17*, *AtPME37* and *AtPME64*) are known to be involved in stress pathways, such as drought, salt and pathogen response [51–53, 66]. Some members of *AtPME*s from groups 2b-1, 4a and 4b-1 are contributed to pollen development [58–64]. Therefore, we predicted that *FvPME* genes of group 1c and 4c-2 might be involved in stress-related pathways, and group 2b-1, 4a and 4b-1 homologues might join in reproductive development. Particularly, in 4a clade, 4a-1 and 4a-3 group members were identified as species-specific subclades. Therefore, we suppose that the Rosaceous *PME* genes from 4a-1 and 4a-3 might develop distinct evolutionary story and functionality which should be verified by further experiment.

### *FvPME38* and *FvPME39* are involved in regulation of fruit softening

**Fig. 5:**
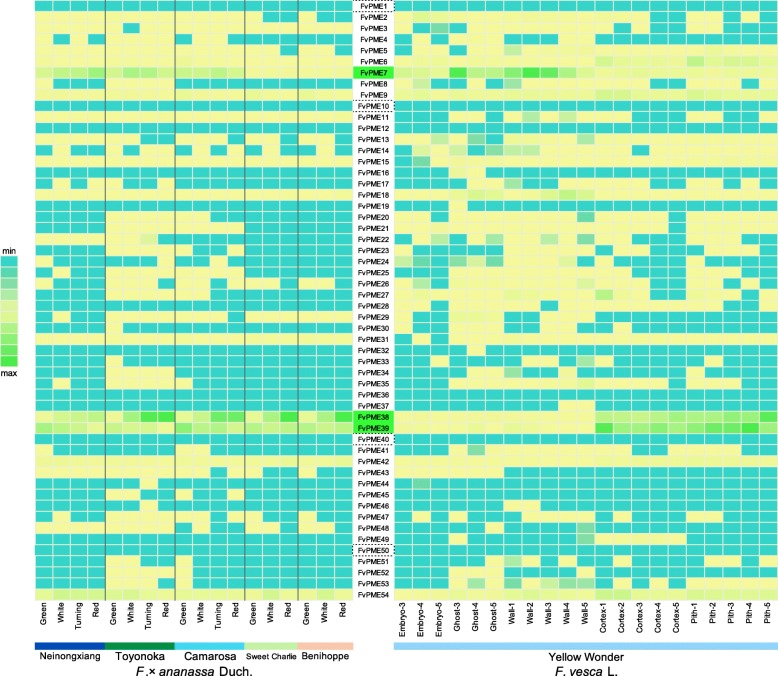
Expression heatmap of strawberry *PME* family genes at developmental stages of fruit. The expression levels of PME genes were measured by RNA-seq analysis at different stages of fruit in five varieties. The color scale at the left represents RPKM normalized by log2. Blue indicates a low expression level, yellow indicates a medium, and green indicates a high level. Four *PME* genes, which are marked by dotted line, don’t have expression level during any developmental stages. And *FvPME 7*, *38*, and *39* expressed abundantly during fruit development which are filled green color

According to the phylogenetic analysis and functional prediction, we aim to study the functionality of FvPMEs during strawberry fruit softening. The spatial and temporal expression patterns of *FvPMEs* showed that *FvPME 7*, *38*, *39*, *42* and *54* were expressed in the strawberry fruit among all *FvPME* members. *FvPME7* was preferentially detectable in embryo, ghost and wall tissues, and *FvPME38* and *FvPME39* had higher expression level in pith and cortex than other tissues (Fig. 5). In line with the previous phylogenetic analysis that the orthologous of *FvPME38*, *AtPME9* has been reported to participate in cell wall remodeling [55]. *FvPME38* and *FvPME39* are good candidates to study PME’s role during strawberry fruit development.

**Fig. 6:**
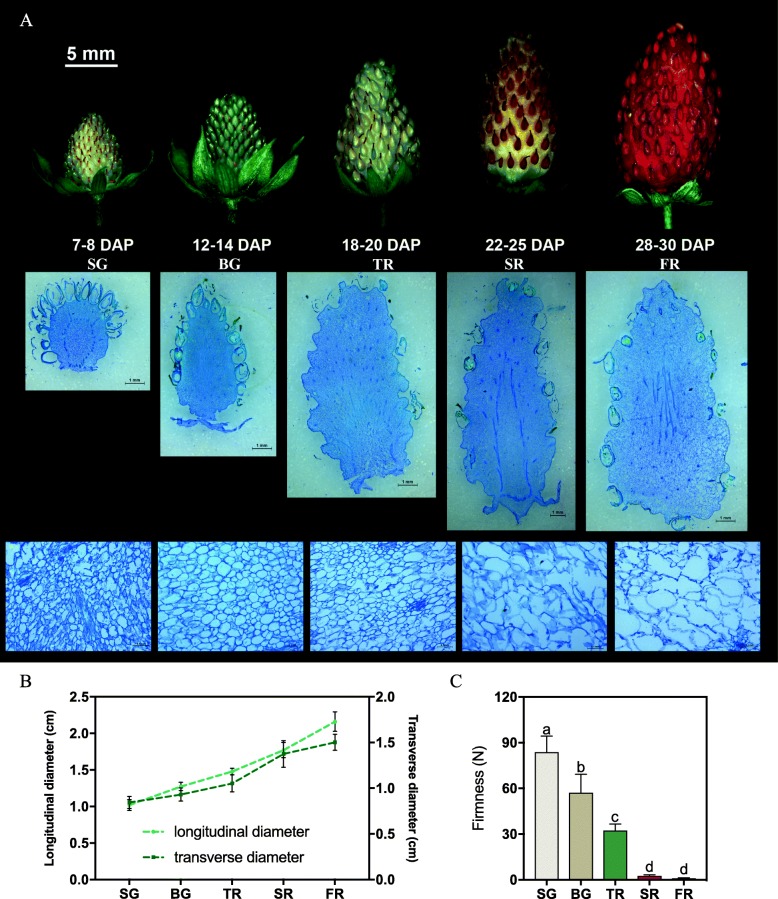
Developmental stages of ‘Ruegen’ fruit. **a**, Five stages of ‘Ruegen’ fruit development. Top row shows each receptacle fruit in different stages. Second row shows longitudinal sections of fruit in five stages. Third row is higher magnifications of sections in second row. **b** and **c**, The changes of diameter and firmness in ‘Ruegen’ fruit during five developmental stages. Values are mean ± SD of ten fruits in (B) and fifteen fruits in (C). DAP, day after pollination. Letter in figure indicates significant differences between groups (*P* < 0.05, one-way ANOVA, Tukey’s HSD post hoc test)

To understand the strawberry fruit developmental process, we firstly conducted morphological and physiological characterization according to the fruit developmental stages. We used diploid woodland strawberry *F. vesca*, ‘Ruegen’ fruit as the study model and divided it into five stages: small green (SG, 7–8 d after pollination [DAP]), big green (BG, 12–14 DAP), turning red (TR, 18–20 DAP), start red (SR, white flesh with red achenes, 22–25 DAP), and full red (FR, 28–30 DAP) stages (Fig. 6a). To examine cell wall structure of fruit, paraffin sections of fruit at different stages were stained by Toluidine Blue O which is commonly employed in polychromatic staining of paraffin embedded plant cell walls [67]. The cell wall texture of the SG, BG and TR stages was more compact than those in SR and FR stages. Moreover, greater cell-cell adhesion and smaller intercellular spaces were present in SG, BG and TR stages, in comparison of larger intercellular spaces with loosely organized cells in SR and FR stages (Fig. 6 a). Along with the gradual expanded fruit size, fruit firmness was significantly decreased from SG to FR stages (Fig. 6 b-c). Particularly, the firmness suddenly became very low level at TR and SR stages (Fig. 6c). The above morphological analysis indicated that fruit swelling and cell wall loosening are probably correlated with the decrease of firmness and tissue integrity during fruit ripening.

**Fig. 7:**
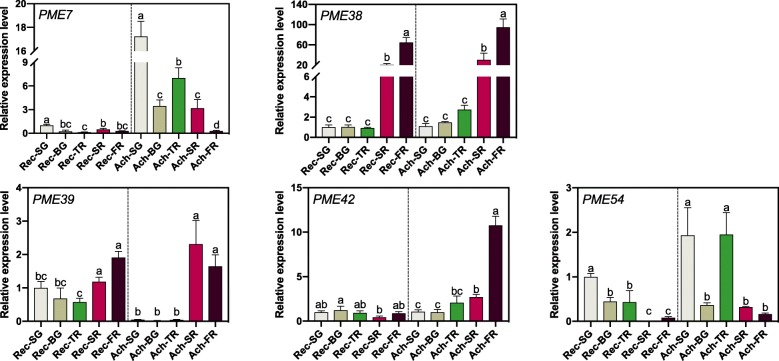
Spatial and temporal expression level of *FvPME* genes during development in achenes and receptacles. Developmental stages were showed in Fig. 6 **a**. Error bars represent SD of three independent replicates. Rec, receptacle; Ach, achene. Letter in figure indicates significant differences between groups (*P* < 0.05, one-way ANOVA, Tukey’s HSD post hoc test)

RNA-seq data showed that *FvPME 7*, *38*, *39*, *42* and *54* were expressed in the strawberry fruit (Fig. 5). The five *FvPME* genes were selected to verify their expression in achenes and receptacles at five stages of fruit development for ‘Ruegen’ by qRT-PCR (Fig. 7). In receptacles, expression of *FvPME7* and *FvPME42* were absent; *FvPME54* expression was detected mainly at early stages, and the expression level of *FvPME38* and *FvPME39* was opposite to that of *FvPME54* which was expressed mainly in ripening stage. In achenes, *FvPME7* was expressed abundantly in early stages, and the expression levels of *FvPME38*, *FvPME39* and *FvPME42* increased at late stages. The expression levels of *FvPME38* and *FvPME39* in receptacles were opposite to the change of fruit firmness which had a sudden drop at SR and FR stages, implying that *FvPME38* and *FvPME39* serve as negative regulators to control fruit rigidity.

**Fig. 8:**
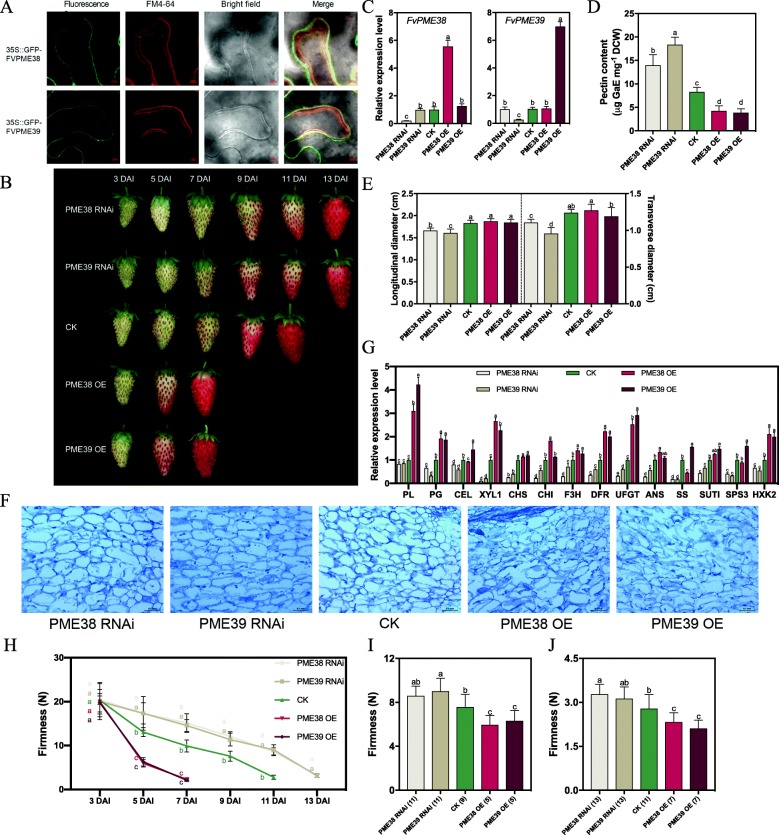
*FvPME38* and *FvPME39* involved in fruit softening. **a**, FvPME38 and FvPME39 were cell wall localized as determined by GFP-FvPME38 and GFP-FvPME39 fusion protein in tobacco leaf epidermal cells. Plasma membrane was identified by FM4–64 staining. **b**, Phenotypes of fruits were agro-infiltrated with *FvPME38* and *FvPME39* overexpression and RNAi constructs, respectively. DAI, day after infiltration; RNAi, RNA interference; OE, overexpression. **c**, QRT-PCR analysis of transcript levels for *FvPME* genes in overexpression and RNAi fruits at three days after injection. Error bars represent SD of three independent replicates. Letter in figure indicates significant differences between groups (*P* < 0.05, one-way ANOVA, Tukey’s HSD post hoc test). **d**-**e**, Pectin content of cell wall (D) and fruit size (E) at seven days after injection. Error bars represent SD of 15 fruits. **f**, Sections of overexpression and RNAi fruits at seven days after injection. **g**, QRT-PCR analysis of transcript levels for ripening-related genes in fruits of transient overexpression or silencing of *FvPME*s. Relative expression levels of each gene were normalized to internal control *Fvactin*. Error bars represent SD of three independent replicates. PL, pectate lyase; PG, polygalacturonase; CEL, cellulose; XYL1, beta-xylosidase1; EXP1, expansin1; CHS, chalcone synthase; CHI, chalconeisomerase; F3H, flavanone 3-hydroxylase; DFR, dihydroflavonol 4-reductase; UFGT, UDP-glucose flavonoid 3-O-glycosyltransferase; ANS, anthocyanidin synthase; SS, sucrose synthase; SUT1, sucrose transporter1; SPS3, sucrose phosphate synthase3; HXK2, hexokinase2. **h**, Texture analysis of fruits were agro-infiltrated with *FvPME38* and *FvPME39* overexpression and RNAi constructs, respectively. **i**-**j**, Firmness value of overexpression and RNAi fruits at same ripening stages after infiltration. SR stage (I); FR stage (J). The number in bracket of x-axis labels represents the number of days after injection. Error bars represent SD of 15 fruits. Letter in figure indicates significant differences between groups (*P* < 0.05, one-way ANOVA, Tukey’s HSD post hoc test)

To examine if FvPME38 and FvPME39 proteins function as pectin-modifying enzymes which should be localized on the cell wall, we transiently expressed *35S::GFP-FvPME38* and *35S::GFP-FvPME39* vectors into tobacco leaves. Green fluorescence signals of *GFP-FvPME38* and *GFP-FvPME39* were detectable specifically in the cell walls which were differentiated from the red fluorescence signals staining by the plasma membrane dye FM4–64 (Fig. 8a).

To further elucidate the roles of *FvPME38* and *FvPME39*, overexpression and RNA interference (RNAi) constructs were agro-infiltrated into ‘Ruegen’ fruit at 18 DAP. After infiltration, we observed a dramatically delay of fruit ripening in *FvPME38* or *FvPME39* RNAi fruits, but *FvPME38* or *FvPME39* overexpressions accelerated ripening process (Fig. 8b and c). *FvPME38* and *FvPME39* RNAi fruits turned full red at 13 days after infiltration (DAI), which is longer than control (11 days), but *FvPME38* and *FvPME39* overexpressing fruits only need 7 days to turn full red (Fig. 8b). Furthermore, in *FvPME38* and *FvPME39* RNAi fruits, pectin content of fruits were significantly higher than the control, and fruits were smaller than the control at 7 DAI; while overexpression of *FvPME38* or *FvPME39* reduced fruit pectin content (Fig. 8d and e). We further examine cell wall texture by paraffin sections combined with Toluidine Blue O staining. The result showed that fruit sections of *FvPME38* or *FvPME39* RNAi exhibited greater cellular adhesion and smaller intercellular spaces, and cell wall structure of parenchymal cell was broken in *FvPME* overexpressors at 7 DAI (Fig. 8f). Beside of the above morphological phenotypes, we also assessed the transcriptional level of downstream genes which indicated fruit ripening, including the softening-related genes polygalacturonase (*PG*), and beta-xylosidase1 (*XYL1*); the anthocyanin biosynthesis genes dihydroflavonol 4-reductase (*DFR*) and UDP-glucose flavonoid 3-O-glycosyltransferase (*UFGT*); and the sugar-related genes hexokinase (*HXK2*) [68–71]. QRT-PCR analysis showed that the transcript levels of *PG*, *XYL1*, *DFR*, *UFGT*, and *HXK2* were significantly downregulated in the *FvPME*-RNAi fruits, but upregulated in the overexpressor fruits, compared with the control (Fig. 8g and Additional file 1: Figure S3). Apparently, FvPME38 and FvPME39 significantly influenced fruit ripening. Texture analysis showed that in *FvPME38* and *FvPME39* RNAi fruits, fruit firmness value was significantly higher than the control fruits at 5, 7, 9, and 11 DAI, respectively (Fig. 8h). And *FvPME38* or *FvPME39* overexpressing fruits were clearly softer than the control at 5 and 7 DAI (Fig. 8h). However, the differences in texture were possibly caused by different ripening stages of fruit. Therefore, we measured the texture of fruit at the same ripening stages (SR and FR stages). The firmness value of *FvPME*-RNAi fruits was significantly higher than that of control, and overexpressing fruits were softer (Fig. 8i and j). Altogether, these results demonstrated that *FvPME38* and *FvPME39* play important roles for regulation of strawberry fruit ripening and softening.

### Abscisic acid regulation of *FvPME 38* and *FvPME 39*

**Fig. 9:**
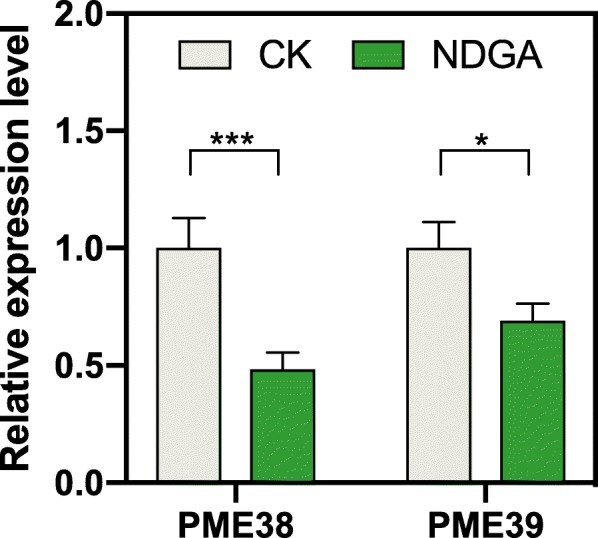
Hormonal regulation of *FvPME*s expression. QRT-PCR analysis of *FvPME38* and *FvPME39* expression in strawberry fruits after 5 days treatment. CK, 15 DAP fruits injected with water; NDGA, 15 DAP fruits injected with NDGA (100 μM). Error bars represent SD of three independent replicates. Asterisk indicates values that were determined by the *t*-test to be significantly different from the control (*, *P* < 0.05; ***, *P* < 0.001)

In previous study, abscisic acid (ABA) has been proved essentially for the onset of ripening process in strawberry fruit [72]. Endogenous ABA content is low at the small white stage and gradually increases through fruit ripening [73], which is consistent with the expression levels of *FvPME 38* and *FvPME 39* genes during receptacle development (Fig. 7). Based on the prediction of the cis-element in *FvPME 38* and *FvPME 39* promoter region, there are some ABRE-elements (Additional file 1: Figure S2 and Additional file 2: Table S5) which belong to the conserved element responding to ABA signal [74]. We speculated that *FvPME 38* and *FvPME 39* may perform downstream of ABA signaling. To verify this hypothesis, we applied nordihydroguaiaretic acid (NDGA), an ABA inhibitor, on the fruits at TR stage to block ABA production. The treated fruits were harvested after five days for qRT-PCR analysis. A significant reduction in the amount of *FvPME 38* and *FvPME 39* transcripts was observed, comparing with the control (Fig. 9). This result indicates that *FvPME 38* and *FvPME 39* expression could be activated by ABA.

## Discussion

### The evolutionary histories of PME have slight differences among rosaceous species

As one of the pectin-modifying enzymes, the functionality of *PME* genes has been widely studied in many plant species, such as *Arabidopsis* [27], rice [28], flax [30], and cotton [31]. Here, 54 *PME* genes were identified in strawberry, and 53, 57, 66, 78 and 79 *PME* genes were found from rose, Chinese plum, peach, apple and pear, respectively (Additional file 2: Table S2). The number of *PME* homologues in Rosaceous species was similar to that in *Arabidopsis,* suggesting a conserved function between Rosaceous plants and *Arabidopsis*. For gene family evolution, a major mechanism to generate new models for evolutionary innovation is the gene duplication, such as tandem gene duplication, WGD/segmental duplication [75]. Following the genome data of six Rosaceous species, all of them experienced one WGD event from one Rosaceous ancestor [76]. And a more recent WGD event could be dated to approximately 40 million years ago in apple and pear, but not in woodland strawberry, rose, peach, and Chinese plum [32, 77–81]. By duplication modes analysis, we found that the tandem duplication events of *PME* gene in peach happen more frequently than those in rose, woodland strawberry and Chinese plum, as shown by the expansion of *PME* gene number in peach than in other species. In apple and pear, WGD/segmental duplication is another main driving force for PME gene family expansion. Therefore, larger numbers of *PME* genes were identified in apple and pear, possibly due to twice WGD events happen in these two species. In addition, *PME* genes of peach only undergo dispersed, tandem and proximal duplications, without WGD/segmental duplication. It suggested that duplication modes of *PME* gene family in those Rosaceous species were diversified. Previous studies have illustrated that a gene family may have common non-random patterns of origin, with conserved duplication modes in different species [82, 83]. However, our results indicated that the main duplication modes of *PME* gene family in the six Rosaceous species were not always strictly conserved, and non-random patterns in different origins were common.

### *FvPME 38* and *FvPME 39* accelerate ripening and softening in fruit

Previous studies have well characterized the general role of PMEs for fruit ripening, whereas PMEs execute different roles in the different plant species. For example, the firmness of *F*. × *ananassa* fruit reduced at the late ripening stages which was concomitant to the high transcript level of *PME* and PG [84]. In different variety of apple, PME activity generally increases during fruit softening; whereas it differed at different stages in different cultivar, which is regulated by ethylene and temperature [26]. Exceptionally, a *PME* gene in apple, called *Malus domestica PME* (*MdPME2),* showed an atypical role [85]. High expression of *MdPME2* in fruit flesh prevents apple fruit mealiness [85]. In some apricot varieties, PME also performs differently in different varieties. ‘San Castrese’ is an apricot variety which maintained the fruit firmness during ripening, and ‘Ceccona’ is another variety which showed rapid softening associated with ripening [86]. During fruit development, PME activity gradually declined in ‘Ceccona’, but it slightly increased in ‘San Castrese’ [86]. Those studies indicated that PME may activate or repress fruit softening in different varieties of same specious, suggesting the various function of PME. The *FvPME38* and *FvPME39* identified in our study displays a typical PME role during fruit ripening, which promotes the softening of strawberry fruits. In previous study, *FaPE1* (AY324809 in the GenBank database), namely *FvPME7* in our study, was specifically expressed in *F*. × *ananassa* fruit, with higher levels during final development stage, coinciding with the beginning of the ripening process [23]. Although our results showed that expression of *FvPME7* was detected mainly at early stages of achenes and clearly absent at all stage of receptacles in *F. vesca* (Fig. 7), which was not coincided with the results of that study. Southern blot analysis showed that *FaPE1* was a single-copy gene in the diploid species *F. vesca*, but elevated allele polymorphism was detectable in the octoploid species *F*. × *ananassa* [23]. It suggested that the number of *FaPE1* allele was extended in *F*. × *ananassa*, which might execute different roles during fruit development between diploid and octoploid strawberry.

Interestingly, beside of the regulation of fruit softening, overexpression of *FvPMEs* accelerates the process of fruit ripening, implying an additional role of FvPME for regulation of fruit ripening. The function of *FvPME38* and *FvPME39* was similar with other cell wall-modifying genes, such as *FvXTH9*, *FvXTH6* and *FaβGAL4*. Overexpression of the genes led to faster ripening by modification of cell wall components in strawberry fruits [87, 88]. Whereas, in tomato fruit, PME activity is only associated with the level of firmness but do not interfere with ripening [24]. Therefore, PMEs in different plant species show diverse functions, which might be caused by the diversity of fruit developmental processes. On the other hand, because of the specific hormonal requirement during fruit ripening, such as climactic fruit and non-climactic fruit [73], PME-mediated pectin degradation and cell wall reconstruction in these two types of fruits might be very different. In our study, *FvPME38* and *FvPME39* had a wide role to affect other genes and overall phenotype. Maybe the *PME*s involved in the modification of some molecular signal. In tomato, an increase of PME activity is associated with a corresponding increase of methanol content during fruit ripening [89]. Methanol is crucial in control of plant growth and response to stresses [90–92]. Pectin demethylesterification by PME is the main source of plant-derived methanol [93]. Therefore, we speculate that cell wall pectin demethylesterification may determine the production of methanol, in turn regulate fruit ripening.

## Conclusions

Our work aims to improve strawberry fruit rigidity by genetic manipulation of key cell wall-modifying enzymes, pectin methylesterases (FvPMEs) during fruit development. Through analysis of the gene evolution in Rosaceous plants, we found that tandem and dispersed duplication events played important roles for the gene expansion of FvPME family. Further genetic manipulation of fruit-specific *FvPME38* and *FvPME39* by overexpression and RNAi-silencing showed that *FvPMEs* significantly influences the fruit firmness, pectin content and cell wall structure, indicating a functional requirement of PME for strawberry fruit softening. These results offer a preliminary understanding of the function and evolution of strawberry *PME* genes, meanwhile provide a knowledge guide for improving fruit firmness by modifying PME level.

## Supplementary information

**Additional file 1: Figure S1.** Logo of conserved motifs in Fig. 2. **Figure S2.** Putative *cis*-elements in the 1.5 kb promoter region of *FvPME*s. **Figure S3.** QRT-PCR analysis of transcript levels of ripening-related genes in fruit of transient overexpression or silencing of *FvPME*s.

**Additional file 2: Table S1.** Primers used for this study. **Table S2.** Pectin methylesterases were identified in *Arabidopsis thaliana*, *Fragaria vesca*, *Malus domestica*, *Pyrus bretschneideri*, *Prunus mume*, *Prunus persica* and *Rosa chinensis*. **Table S3.** Conserved domains were predicted by CDD. **Table S4.** Gene duplication modes of PME genes in *Arabidopsis thaliana*, *Fragaria vesca*, *Malus domestica*, *Pyrus bretschneideri*, *Prunus mume*, *Prunus persica* and *Rosa chinensis*. **Table S5.** Cis-elements were predicted by PlantCARE in promoter sequences of *FvPME* genes.

## Data Availability

All Arabidopsis protein sequences were downloaded from The Arabidopsis Information Resource (TAIR) (https:// www.arabidopsis.org). The gene files of apple, strawberry, Chinese plum, peach and rose were downloaded from GDR database (Genome Database for Rosaceae: http://www.rosaceae.org/). The gene files of pear were downloaded from the pear genome database (http://peargenome.njau.edu.cn/). The RNA-Seq data of different strawberry varieties was obtained from NCBI (Neinongxiang, PRJNA438551; Toyonoka, PRJNA394190; Camarosa, PRJEB12420; Sweet Charlie, PRJNA263114; Benihoppe, PRJNA473417; Yellow wonder, SRA065786).

## Abbreviations

ABA: Abscisic acid
ANS: Anthocyanidin synthase
BG: Big green stage
BLAST: The basic local alignment search tool
CEL: Cellulose
CHI: Chalconeisomerase
CHS: Chalcone synthase
DAI: Day after infiltration
DAP: Day after pollination
DFR: Dihydroflavonol 4-reductase
EXP1: Expansin1
F3H: Flavanone 3-hydroxylase
FR: Full red stage
HG: Homogalacturonan
HMM: Hidden markov model
HXK2: Hexokinase2
MEGA: Molecular evolutionary genetics analysis
MEME: Multiple em for motif elicitation
ML: Maximum likelihood
NDGA: Nordihydroguaiaretic acid
NJ: Neighbor-joining
OE: Overexpression
Pfam: Protein family
PG: Polygalacturonase
PL: Pectate lyase
PME: Pectin methylesterase
QRT-PCR: Quantitative reverse transcription–PCR
RNAi: RNA interference
RNA-seq: Ribose nucleic acid sequencing
RPKM: Reads per kilobase per million reads
SG: Small green stage
SMART: A simple modular architecture research tool
SPS3: Sucrose phosphate synthase3
SR: Start red stage
SS: Sucrose synthase
SUT1: Sucrose transporter1
TR: Turning red stage
UFGT: UDP-glucose flavonoid 3-O-glycosyltransferase
WGD: Whole-genome duplication
XYL1: Beta-xylosidase1
