# Supplementary material for: Genome wide identification and functional characterization of strawberry pectin methylesterases related to fruit softening
Source: BMC Plant Biol. 2020 Jan 8;20:13. doi: 10.1186/s12870-019-2225-9 (PMC6950920; doi:10.1186/s12870-019-2225-9)
Supplement: Supplementary file 1 — Additional file 1: Figure S1. Logo of conserved motifs in Fig. 2. Figure S2. Putative cis-elements in the 1.5 kb promoter region of FvPMEs. Figure S3. QRT-PCR analysis of transcript levels of ripening-related genes in fruit of transient overexpression or silencing of FvPMEs. [file 12870_2019_2225_MOESM1_ESM.pdf]

Supplemental figure 1-3

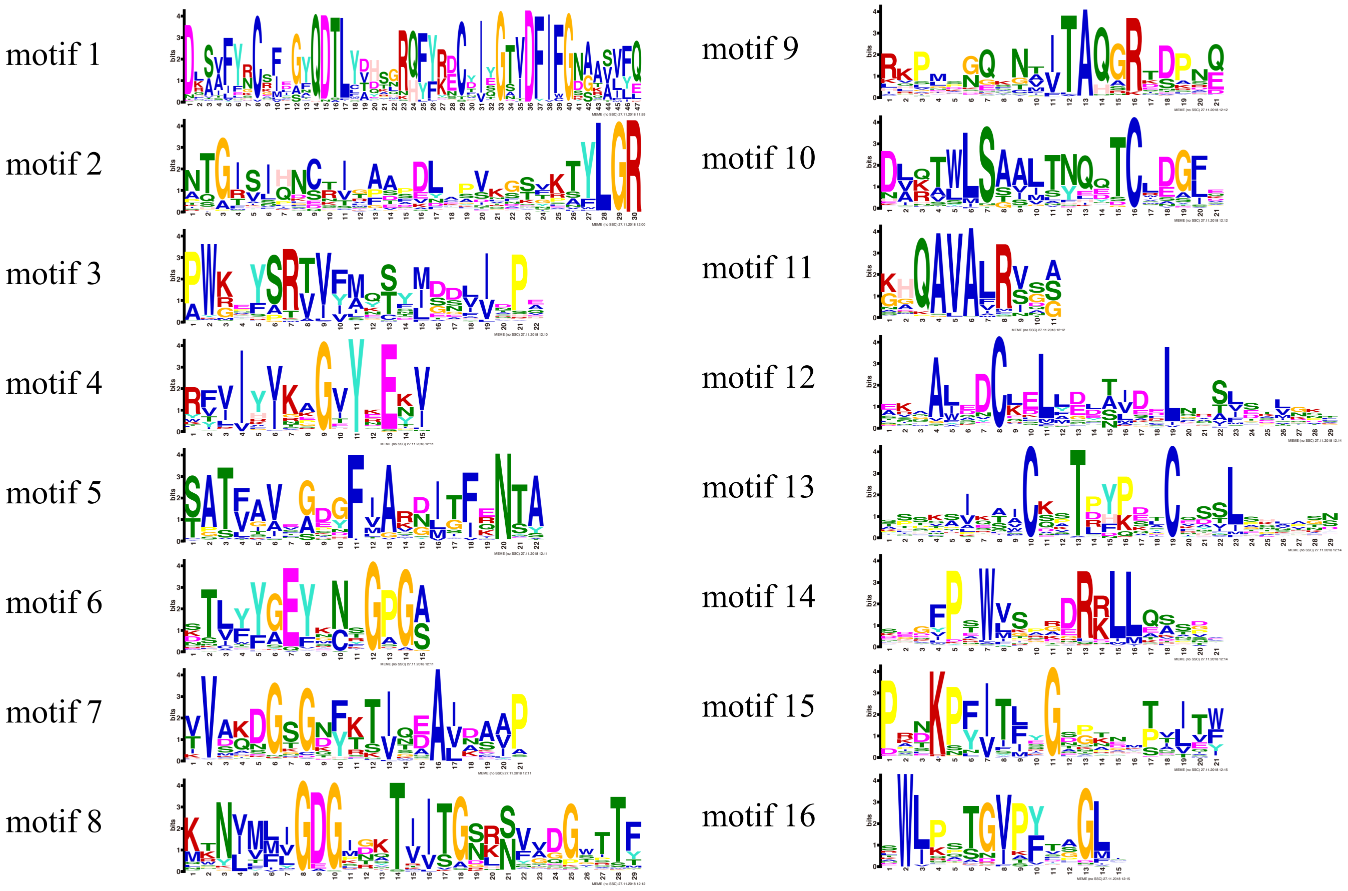

Figure S1 Logo of conserved motifs in Figure 3.

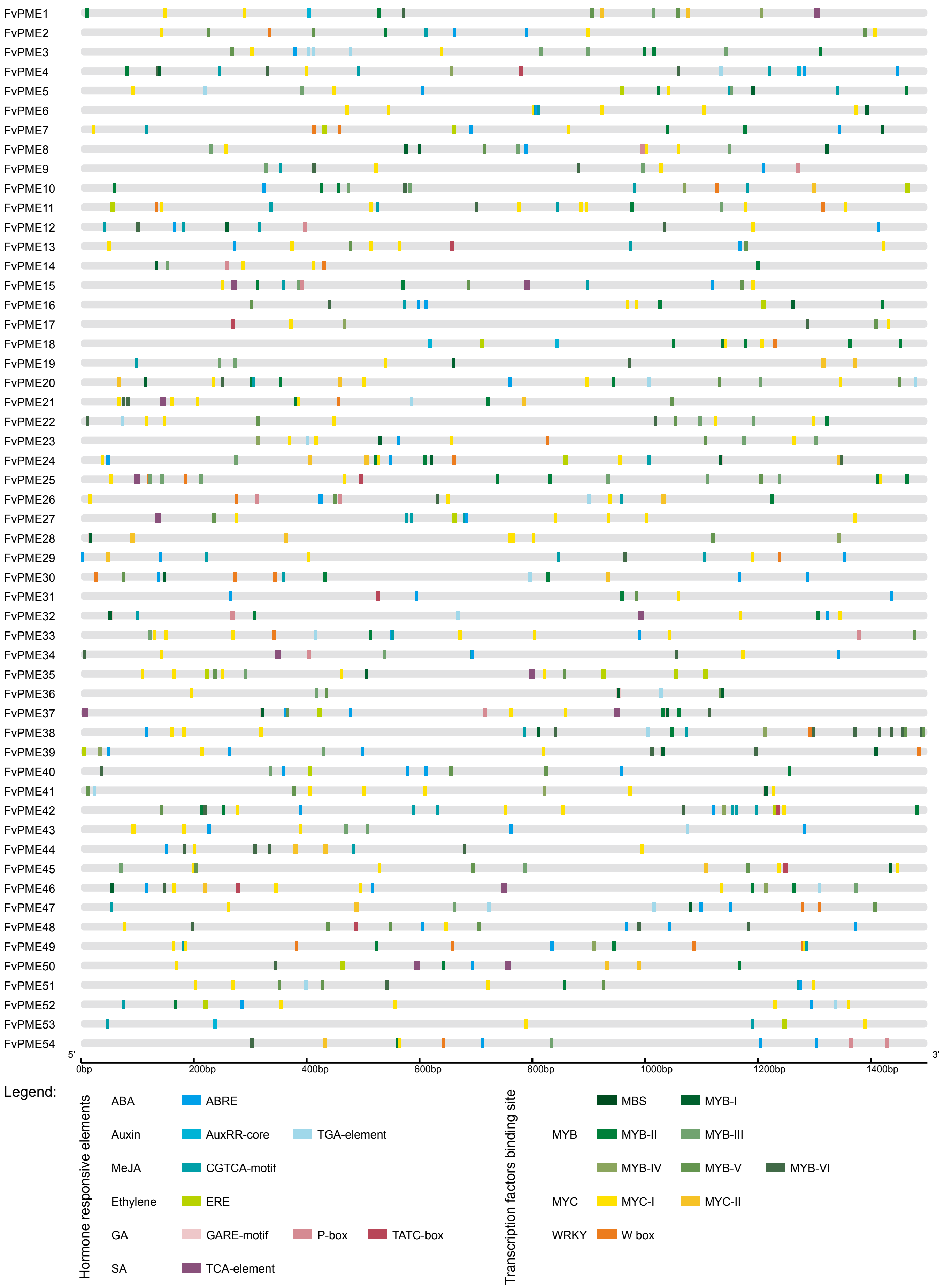

Figure S2 Putative cis-elements in the 1.5 kb promoter region of FvPMEs. Those cis-elements could be distinguished from each other with different colour, and their relative position are displayed.

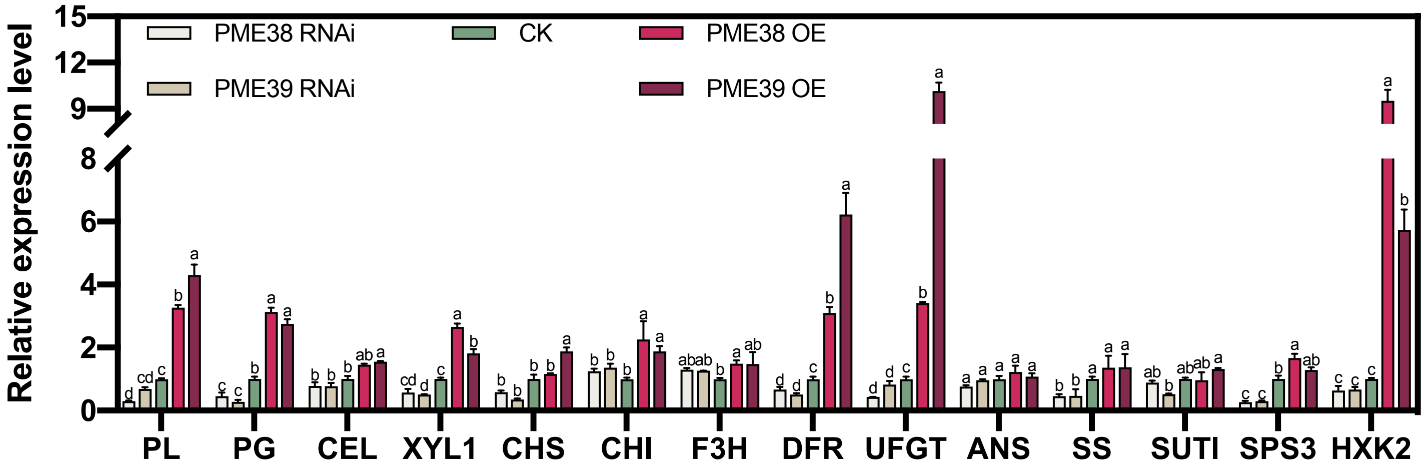

Figure S3 QRT-PCR analysis of transcript levels for ripening-related genes in fruits of transient overexpression or silencing of *FvPME*. Error bars represent SD of three independent replicates. Relative expression levels of each gene were normalized to internal control *FvGAPDH*.
